# Evaluating the Usage of Musculoskeletal Spinal Drop-In Services in East Lancashire: A Retrospective Audit of Performance Standards

**DOI:** 10.7759/cureus.43543

**Published:** 2023-08-15

**Authors:** Khalid A Al-Hashimi, Umar N Said, Abdullah Elrefae, Taherah Khan

**Affiliations:** 1 Vascular Surgery, Colchester Hospital, Colchester, GBR; 2 Trauma and Orthopedics, Huddersfield Royal Infirmary, Huddersfield, GBR; 3 Breast Surgery, Colchester Hospital, Colchester, GBR; 4 Medical Education, Worcestershire Acute Hospitals NHS Trust, Worcester, GBR

**Keywords:** clincal audit, walk in clinic, msk pain, start back tool, lower back pain (lbp)

## Abstract

Lower back pain (LBP) is a prevalent musculoskeletal disorder (MSD) that places a significant burden on patients as well as healthcare and economic systems. Musculoskeletal (MSK) spinal drop-in clinics in the North West of the United Kingdom (UK) have been introduced to provide more targeted therapies for those suffering from LBP. A retrospective audit was conducted from January to February 2017 to evaluate the utilization of the spinal clinic in relation to individual patient Keele STarT Back prognostication scores and to compare these with national guidelines. A total of 50 patients’ case notes were reviewed over the four-week period. The focus was placed on how patients were made aware of the clinic, whether they had been seen by a primary care provider, and if first-line therapies had been administered. The results of this study demonstrate that some improvement is required in patient management and seek to provide recommendations for optimizing the service.

## Introduction

Musculoskeletal disorders (MSDs) are described as strenuous pains within the musculoskeletal (MSK) system; they comprise various pathologies affecting bones, joints, muscles, and connective tissues [1]. Globally, they are the leading source of pain [2], and estimates reveal MSDs to be one of the most common causes of primary care visits, with up to a fifth of the adult population presenting to a general practitioner (GP) each year [3, 4]. MSDs are highly variable in presentation and range from being self-limiting in nature to impinging greatly on patient mobility as well as the ability to perform their activities of daily living (ADL). Lower back pain (LBP) is the most prevalent of all MSDs [5-7], and the WHO recognizes it as a leading contributor to disability development worldwide [8]. Epidemiological data estimates the current prevalence of LBP to exceed over 500 million, affecting up to 33% of the global population at any one point in time; this has increased significantly within the last two decades [9, 10]. In earlier years, the burden imposed by back pain was characterized as insignificant [5], though recent evidence suggests otherwise. MSDs place significant strain on sufferers, healthcare, and social support systems, with substantial economic ramifications [11]. Back pain has historically cost the UK economy over £10 billion annually [12]. National reports have noted that the average amount of time lost in employment due to back pain approximates 15.2 days per case, with a total of 7.3 million days of work lost each year by the UK workforce [13]. Additionally, individuals struggling with prolonged periods of debilitating back pain have demonstrated an increased frequency of recurrent absence and a reduced likelihood of returning to work altogether [14]. Despite increased expenditure for LBP and the introduction of a plethora of treatments, rates of back-related disability have increased while patient outcomes remain largely variable [15, 16, 17]. Traditionally, opioids have been the mainstay of symptomatic control. However, consensus shows they pose no benefit in improving the functional outcomes of these patients [15]. The detriments associated with their overuse include long-term dependency and mortality risk secondary to respiratory depression. Consequentially, this has resulted in integrating psychosocial interventions into an initially thought biomedical problem [18]. Delivery of early effective management of the correct intensity requires the implementation of a standardized approach. The National Institute for Health and Care Excellence (NICE) has recognized the utilization of risk stratification tools in aiding the initial and long-term management of all new episodes of LBP [19]. National guidance recommends the Keele STarT Back Screening Tool in the risk stratification within this patient group. Once patients are admitted for each new episode of back pain, the scoring system provides a systematic approach allowing for grouping into 'low risk,' ‘medium risk,' and 'high risk' subgroups based on their score. Low-risk patients score less than 4 points on the tool, while medium-risk patients score 4 or more points, with 3 or less of these points being in questions 5-9 of the tool. High-risk patients score 4 or more points, with 4 or more points distributed in questions 5-9. This, in turn, leads to the identification of those with a higher possibility of future disability in order to receive the necessary matched treatment [20].

MSK spinal drop-in clinics are currently employed within the United Kingdom (UK). They were introduced to aid in the assessment and community-based management of neck and back pain, with or without limb involvement, in those aged 16 and over. Benefits of their use include improved access to timely care while reducing the burden of avoidable presentations to the ED. The clinics are utilized as a walk-in service, though patients are advised to consult their GP prior to attendance. Current practice involves patient completion of the Keele STarT Back screening tool followed by an initial hour-long assessment by a general physiotherapist. Those with particularly complex needs underwent assessment by an extended-scope physiotherapist or a GP with a known special interest in MSK health. This study aimed to evaluate patient utilization of the spinal drop-in clinics in line with local advice while also assessing any barriers patients may face while seeking care. Moreover, we intended to assess current performance standards against NICE's NG59 national guidance relating to the use of risk stratification tools and the provision of appropriate analgesia.

## Materials and methods

**Figure 1:**
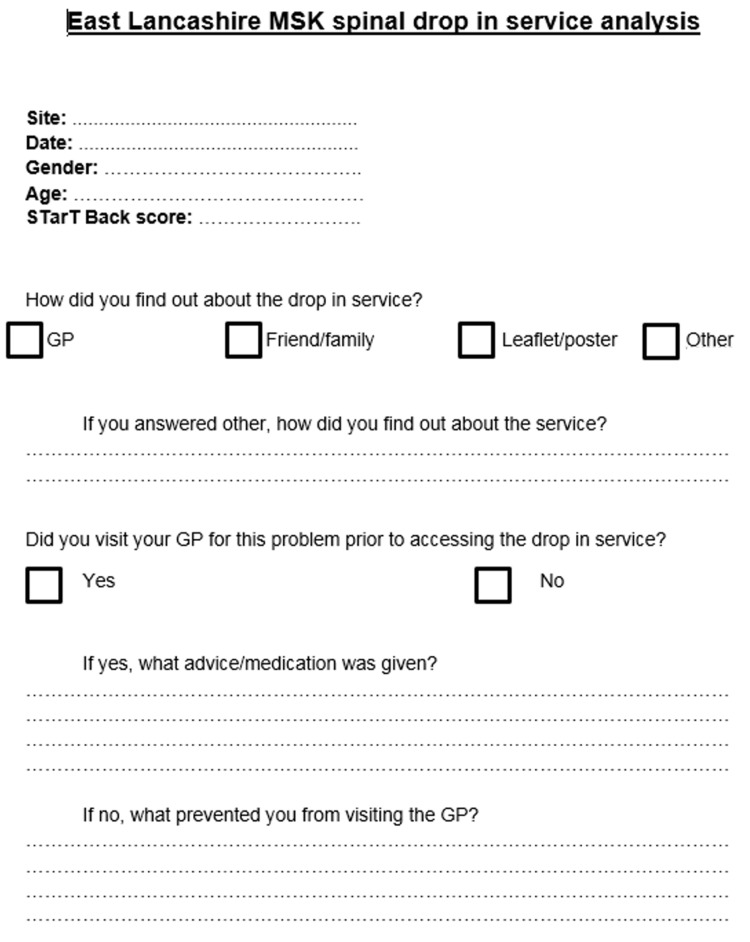
Spinal drop-in service analysis study questionnaire.

The study was performed between January and February 2017. We aimed to evaluate the service by identifying how patients were made aware of the drop-in service and whether patients were being managed appropriately in a primary care setting. Each patient admitted to the spinal drop-in clinic was asked by a non-clinical staff member to complete a proforma with the Keele STarT Back questionnaire on initial presentation to the clinic. Their score was used for subsequent classification into their respective stratified risk group in an attempt to ensure they received the appropriate level of treatment. A second questionnaire (Figure 1) was developed in line with the study's objectives; this included a section highlighting the patient's STarT Back score. All first-encounter patients who had visited within the period set were eligible to be included in the study; only those who had previously been seen within the spinal drop-in clinic were excluded. A total of 63 patients were identified throughout the four weeks and asked to complete both questionnaires. Of the 63 responses, 12 were identified as incorrectly completed by the patients and thus were removed from the study. Fifty patients consented to be included. One refused to partake in the cross-sectional study. The proforma focused specifically on the channels by which the patients presented to the clinic. Moreover, other variables of interest included their respective STarT Back scores and whether they had received consultation within the community. If these patients were not seen prior to presentation to the drop-in service, the questionnaire allowed us to identify the key barriers they faced in accessing primary care. If patients had been seen by the GP, information on provision of pharmacological therapies was also collected to assess current performance against NICE guidance.

## Results

This cross-sectional study received responses from 62 of the original 63 participants (a response rate of 98%) who utilized the service between January and February 2017. However, 12 of these responses were excluded because the questionnaires were not completed properly. Thus, 50 valid questionnaires were included for the period between January and February 2017. Of these, 22 (44%) were from males and 28 (56%) were from females. The average age of patients presenting to spinal clinics was 46.8 years, ranging from 22 to 75 years old. As Table 1 shows, the majority of patients using the MSK drop-in service in the East Lancashire region were in the 30-39-year-old age group, accounting for 26%. The age ranges of 40-49, 50-59, and 60-69 had similar percentages of use at 18%, 22%, and 20%, respectively. Extremes of age formed the minority of presenting patients; no patients below the age of 22 presented to the clinic, and those aged 70 and above constituted 4% of presentations. Despite the anticipation of smaller populations of younger patients presenting to the MSK, a larger population of elderly patients was anticipated within the sample set.

**Table 1:** Age distribution of patients presenting to the East Lancashire MSK spinal drop-in service between January and February 2017. MSK: Musculoskeletal.

Age	Number	Percentage (%)
0-18	0	0
19-29	5	10
30-39	13	26
40-49	9	18
50-59	11	22
60-69	10	20
70+	2	4

Within the study, we collected additional data on the different methods through which patients were introduced to the clinic service, along with each patient's corresponding STaRT back score. Each of the participants was able to select one of the four possible options by which they were made aware of the MSK spinal clinics: ‘via their GP,’ ‘friends or family,’ ‘leaflet or poster,’ and ‘other.’ A total of 43 of the 50 (86%) were either directly referred or received a recommendation by their GP, 4 (8%) following a recommendation by friends or family, one (2%) from a leaflet or poster, and two (4%) by other additional means. One of the patients worked directly for the service itself, while the other worked within the same building as a spinal clinic service. The overall average STarT back score was 5.46 (range 1-9), with the highest average of 6 in those in the age group greater than 70 years. The lowest average STarT back score was seen in those aged between 40-49 at 4.7 (Table 2). Among the patients who underwent GP referral, sub-group analysis revealed that the most common STarT back scores were both 3 and 6, while patients with scores of 2 presented less frequently to the MSK clinics (Table 3).

**Table 2:** STarT Back Score distributions of patients presenting to East Lancashire MSK spinal drop-in services between January and February 2017. MSK: Musculoskeletal.

Age	Average STarT Back Score	Range of STarT Back Scores
0-18	0.0	0
19-29	5.8	4-8
30-39	5.5	1-9
40-49	4.7	2-9
50-59	5.4	3-9
60-69	5.8	2-9
70+	6.0	4-8

**Table 3:** Number of patients presenting to East Lancashire MSK spinal drop-in services between January and February 2017 by STarT Back score (of the 43 patients referred to the service via their GP). MSK: Musculoskeletal.

STarT back score	Number of patients
1	0
2	3
3	7
4	4
5	6
6	7
7	5
8	5
9	6

Furthermore, the questionnaire filled out by the 50 patients facilitated data collection concerning treatments initiated by GP practices before patients' consultations in the spinal clinics. Findings revealed that 21 of the 43 patients (49%) had received no form of first-line treatment by their GP before referral. However, it was noted that two of the following 21 patients had received recommendations to visit one of the clinics without being seen directly within the community. The remaining 22 (51%) received intervention within the primary care setting (Table 4); all patients received some form of pharmacological therapy. These include paracetamol, non-steroidal anti-inflammatory drugs (NSAIDs) with or without weak opioids, tricyclic anti-depressants (TCAs), strong opioids, or a combination of various analgesic medications.

**Table 4:** Distribution of pharmacological treatments provided to patients by community GPs. GP: General practitioner; NSAIDs: Non-steroidal anti-inflammatory drugs; TCA: Tricyclic anti-depressants.

Highest level of drug treatment	Number of patients
Paracetamol	4
NSAIDs/weak opioids	12
TCA’s	1
Strong opioids	1
Combination of drugs	4

In the patient category receiving NSAIDs with or without weak opioid preparations, all 12 patients were prescribed either naproxen or ibuprofen by their GP. The drug cards of these patients were checked for gastric protection with a proton pump inhibitor (PPI) in line with NICE guidance. Of the 12, 10 (83%) were found to not have been co-prescribed a PPI at the time. Seven patients were not referred by their GP to the service. Six patients revealed that the main barrier they encountered was accessing their respective GP practices; one of the seven patients admitted to directly accessing the spinal clinic due to ongoing pain-related concerns and expressed concerns regarding waiting times for a GP appointment. Of the six patients who faced difficulty accessing their GP practices, prolonged wait times on phone lines were reported by all patients as the reason for the inability to book an appointment with their primary care physicians.

## Discussion

A study by Hill JC et al. in 2016 demonstrated that a stratified approach to managing back pain in primary care is best achieved through the provision of multi-faceted input where necessary through categorization into low, medium, and high-risk groups; those who qualify into medium and higher risk groups are more likely to be offered cognitive behavioral therapies (CBT) as well as intensive physiotherapy input [20]. Although only one month of data collection took place, specific points for quality improvement were clearly identified. A total of 22 patients presenting at the spinal drop-in services within East Lancashire were male (44%), while 28 (56%) were female. This corresponds with the greater prevalence of back pain expected within female populations [21, 22] and has been attributed to various influences. Bio-psychosocial models have been employed to explain differences in pain between sexes by focusing on biological, psychological, and social factors. Biological explanations for increased back pain within female populations include a gradual loss of osteo-protection secondary to a decline in hormones such as estrogen, especially throughout menopause [23]. The average age of patients was 46.8 years, with an age range of 22-75 years. Females presenting to the drop-in service had an average age of 48.8 years, while the average age for males was 44.3 years. Results showed that the service is utilized fairly equally across most age groups. However, those aged 0-18 and 70+ were under-represented; no patients below 18 years were surveyed, and only two patients (4%) aged 70 or older responded during this audit. One possible explanation is that these age groups may have more complex presentations, such as secondary neurological involvement, requiring specialized care in a secondary setting. Pediatric patients with spinal disorders, like scoliosis or kyphosis, need immediate imaging and specialist input. Similarly, elderly patients with severe neurological deficits require rapid assessments. The average STarT back score of the 43 patients who were referred to the spinal drop-in service by their GP was calculated to be 5.56, indicating that the average person referred to the service is most likely to be either medium or high risk as per the STarT back screening tools guidelines. Patients scoring 3 or less on the STarT back tool are deemed as low risks; however, a total of 10 patients were still unnecessarily referred to a local MSK spinal clinic. Some concerns also arose from the fact that only 22 (51%) of the 43 referred to the service by their GPs had received some form of analgesia; despite this, it remains a possibility that some patients with lower scores may have used over the counter preparations of paracetamol or ibuprofen. 

Though it is likely that the spinal service has provided great support to patients within the community, the following represents only a small subset of the population as the data collected for this study was limited over the course of a single month. Thus, limited by smaller numbers and short periods indicates that a larger scale study is required to provide more valid conclusions. Findings suggest that a significant proportion of patients completely lacked any form of early management. The data demonstrates that 14% of patients were not seen prior to primary care. The key barrier experienced by patients was the inability to obtain an appointment with their GPs due to prolonged wait times over the phone while attempting to book. Moreover, of those who were seen within primary care, approximately half of the patients were not started on first-line analgesic treatment. This could be confounded by other factors, such as patients using over-the-counter analgesia. However, it may also suggest that a review of back pain management within primary care might be beneficial, especially in targeted practices where initial treatment wasn't started upon presentation. Such practices could be identified using the data from the 29 patients and their medical records. The data collected for this study indicated that none of the patients prescribed NSAIDs were also given a PPI, as per current guidance. A potential method to determine if this trend represents primary care prescribing within the UK could be the basis for an audit. This could be conducted across one or multiple GP practices, focusing on anyone flagged in the system as receiving NSAID therapy for back pain. Checking to see if PPIs are co-prescribed would be simple due to modern electronic patient records. A more recent re-audit of patient presentations to the MSK spinal drop-in clinics within East Lancashire over the course of 12 months would provide further data. Doing so would increase the confidence in the above recommendations. Further data collected over the course of 12 months would increase the confidence in the above recommendations and may provide further in-depth information regarding the management of LBP within different geographical locations within East Lancashire, showing where additional training related to early pain management could be sent to primary care centers. Considerations must be made regarding the impact of the COVID-19 pandemic, as this study was conducted in the pre-pandemic era. A more recent study could then be presented to clinical commissioning groups (CCGs) for comparison, to assess differences in performance post-pandemic.

## Conclusions

In conclusion, this study has highlighted areas for improvement that exist in the management of LBP patients within the East Lancashire region. The introduction of MSK spinal drop-in clinics aimed to provide targeted therapies for LBP in the United Kingdom (UK); recommendations for service optimization include ensuring appropriate referral of patients based on risk scores and improving early management of LBP within primary care, including both access to GP services as well as pharmacological alleviation of symptoms. Further re-auditing of performance over a more prolonged period is necessary post-pandemic to assess the long-term impact of interventions and to identify additional areas for improvement in LBP management.
